# Ultrasound and intestinal lesions in *Schistosoma mansoni* infection: A case-control pilot study outside endemic areas

**DOI:** 10.1371/journal.pone.0209333

**Published:** 2018-12-18

**Authors:** Francesca Tamarozzi, Dora Buonfrate, Geraldo Badona Monteiro, Joachim Richter, Federico Giovanni Gobbi, Zeno Bisoffi

**Affiliations:** 1 Centre for Tropical Diseases, IRCCS Sacro Cuore-Don Calabria Hospital, Negrar, Italy; 2 Institute of Tropical Medicine and International Health, Universitäts-Medizin, Berlin, Germany; 3 Diagnostic and Public Health Department, Infectious Diseases and Tropical Medicine Section, University of Verona, Verona, Italy; George Washington University, UNITED STATES

## Abstract

**Background:**

Infection with *Schistosoma mansoni* is a major cause of morbidity and mortality in endemic areas, and is increasingly diagnosed in migrants and travellers outside transmission areas. Markers for the assessment of morbidity and impact of control programs in endemic areas and for the clinical management of patients in the clinical setting are scant, especially for intestinal involvement. Ultrasonography is well established to evaluate hepatosplenic pathology; on the contrary, ultrasound evaluation of intestinal schistosomiasis is virtually unexplored. In this pilot study, we aimed to describe and evaluate the accuracy of unenhanced intestinal ultrasound for morbidity due to intestinal *S*. *mansoni* infection.

**Methodology/Principal findings:**

We performed a blind case-control study of unenhanced intestinal ultrasound on 107 adults accessing the outpatient clinic of our Centre for Tropical Diseases between January-July 2018 as part of a screening for tropical diseases in migrants and travellers returning from endemic areas. Other clinical and laboratory data were obtained routine examination reports. We could not find any overtly pathological thickness of the gut wall in the sigma, proximal ascending colon, and terminal ileum, in patients with *S*. *mansoni* infection (n = 17), *S*. *haematobium* infection (n = 7), positive anti-Schistosoma serology (n = 31), and uninfected individuals (n = 52), with no difference among groups as assessed by ANOVA. No polyps or other intestinal abnormalities were visualized. There was no significant change in gut wall thickness one month after treatment with praziquantel in patients with *S*. *mansoni* infection (n = 11).

**Conclusions/Significance:**

Our preliminary results suggest that intestinal ultrasound might not be a sensitive tool for detecting minor intestinal morbidity due to schistosomiasis. Further studies in a hospital setting comparing colonoscopy and ultrasonography may be envisaged; in endemic areas, further studies are needed to describe and assess the usefulness of intestinal ultrasound in patients stratified by infection intensity and compared with markers such as calprotectin and fecal occult blood.

## Introduction

Schistosomiasis is a parasitic infection caused by trematodes of the genus *Schistosoma*, acquired upon contact with fresh water bodies contaminated with the larval stage of the parasite. It is estimated that more than 200 million people are infected worldwide by schistosomes causing either the urinary or the intestinal form of the disease, resulting in over 200 thousand deaths every year, mostly in Sub-Saharan Africa [1, 2]. Imported schistosomiasis in migrants and travellers returning from endemic areas has been defined, in receiving countries such as Italy, as a “hidden epidemic”[3].

*S*. *mansoni*, the main causative agent of intestinal and hepatosplenic schistosomiasis, is estimated to infect over 50 million people in endemic areas [4]. Pathology is caused by eggs released by female parasites residing in the venous plexus draining the large intestine, which are trapped in the bowel wall and the liver [5]. In recent years, there has been considerable effort by the international community toward the control of the infection with mass drug administration of praziquantel [1]. The aim of this strategy is the decrease in morbidity due to schistosomiasis [6]. However, morbidity markers for assessment of the infection and post-interventions evaluation are scant [7]. Also at an individual patient’s level, a comprehensive evaluation of morbidity is difficult due to the variability of schistosomiasis-related clinical manifestations and lack of specific symptoms.

Morbidity and mortality due to advanced hepatosplenic involvement resulting in liver fibrosis and portal hypertension are well recognized and described [4, 8]. On the contrary, morbidity due to involvement of the bowel, which is affected by the inflammatory response to eggs passing through and entrapped in the mucosa and sub-mucosa, has proven more difficult to quantify. This is partly due to the variability and lack of specificity of symptoms, which depend on the extent of inflammation and range from vague abdominal pain to bloody diarrhoea. Based on questionnaires data, diarrhoea with or without blood has been estimated to occur in over 5 million individuals infected with *S*. *mansoni* in sub-Saharan Africa, but this estimate is burdened by high uncertainty [4]. The clinical diagnostic gold standard for intestinal morbidity is colonoscopy, but this has virtually no applicability for disease burden evaluation at population level and is also poorly accepted and repeatable in asymptomatic or pauci-symptomatic individuals in the clinical setting. Faecal calprotectin and faecal occult blood, unspecific markers of bowel inflammation, have been recently shown to correlate with parasite egg excretion and therefore suggested to be of use for monitoring in control programs [9].

Ultrasonography (US) is a particularly appealing tool in the field of infectious diseases because it is non-invasive, devoid of radiations, repeatable, and can be performed at the bedside of the patient with the use of portable machines [10]. Even in case of unspecific findings, the visualization of the affected organ, complementary to what provided by laboratory tests, provides useful information on the type and extent of pathology. In *S*. *mansoni* infection, US is a well-established technique to evaluate hepatosplenic pathology [10, 11]. A standardized protocol for the evaluation and grading of hepatosplenic schistosomiasis using US was issued by the WHO in 2000 [12] and recently evaluated for usability and usefulness [6]. On the contrary, reports on the use of US in the evaluation of intestinal schistosomiasis are extremely scant [11, 13], and there is no US report on the evolution of the lesions, when visualized, after treatment, either in endemic areas or in case of no risk of reinfection.

There is a need to find low-cost, reliable, and repeatable markers of bowel morbidity that can aid both the assessment of morbidity and impact of control programs in endemic areas and the clinical evaluation and follow-up of individual patients in the clinical setting. The primary objective of this study was to describe and evaluate the accuracy of unenhanced intestinal US signs for the diagnosis and morbidity assessment of intestinal *S*. *mansoni* infection. The secondary objective was to evaluate the evolution over time of unenhanced intestinal US signs in patients with *S*. *mansoni* infection.

## Methods

### Ethics statement

The study was approved by the Ethics Committee for Clinical Experimentation of Verona and Rovigo Provinces, Italy (protocol n. 63537). All adult participants provided written informed consent; children were excluded from participation.

### Study design and participants

**Fig 1:**
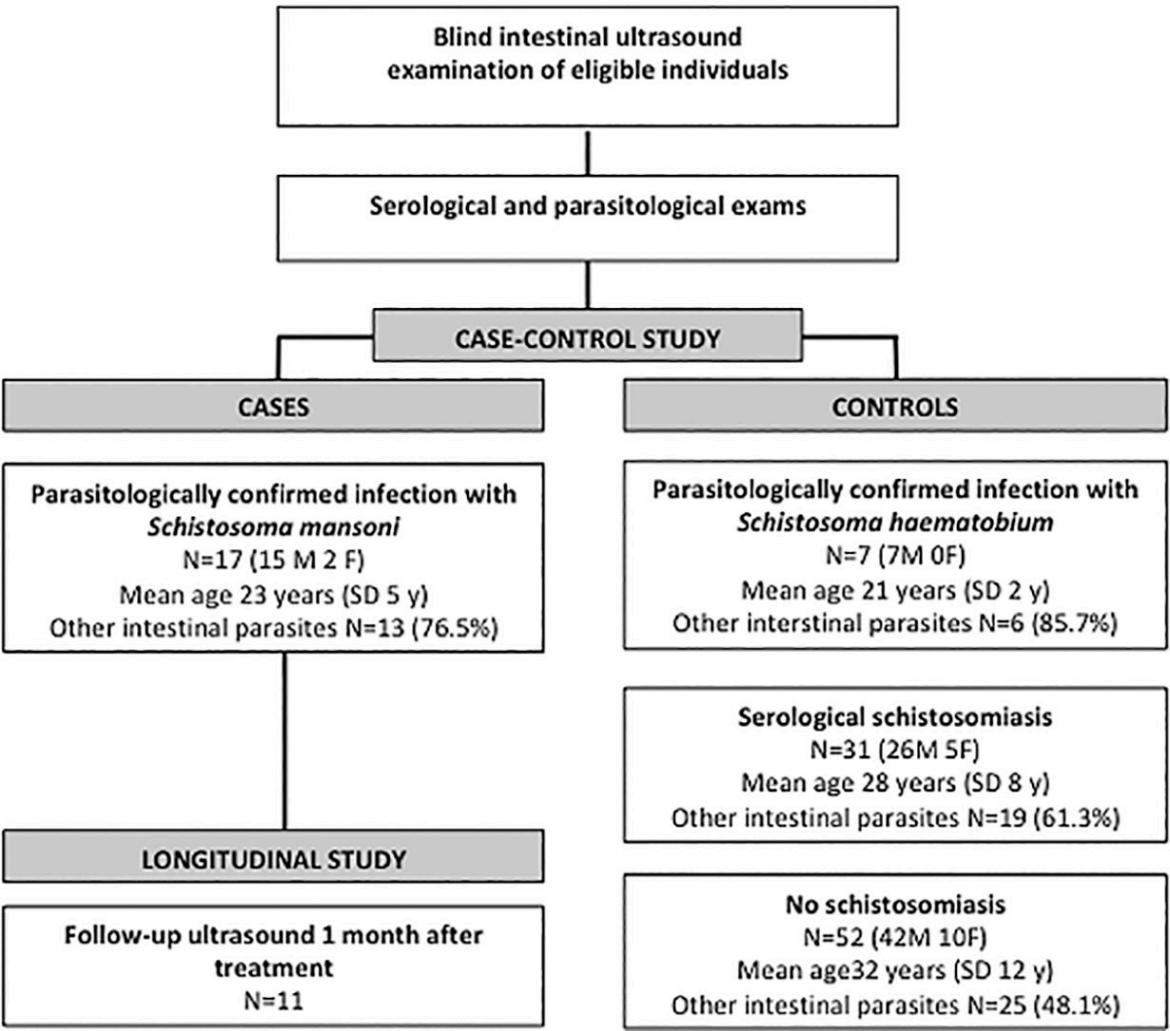
Study flowchart and demographic and clinical characteristics of enrolled subjects at first visit. M = males; F = females; SD = Standard Deviation.

This is a case-control, cross-sectional pilot study followed by a longitudinal study of unenhanced intestinal US (Fig 1). The study was carried out on a convenience sample of asymptomatic/pauci-symptomatic individuals accessing the outpatient clinic of the Centre tor Tropical Diseases of the IRCCS Sacro Cuore-Don Calabria Hospital of Negrar, Verona, Italy, between January 1^st^ and July 1^st^ 2018 as part of a screening for tropical diseases in migrants and travellers returning from endemic areas.

Inclusion criteria for the case-control study were: epidemiological risk for *S*. *mansoni* infection, age ≥ 18 years, and no documented evidence of receiving treatment with praziquantel in the previous 6 months. This period was decided based on the known behaviour on US of bladder lesions due to *S*. *haematobium*, which resolve completely by 6 months post-treatment (in the absence of neoplastic transformation) but could be still detectable within this time interval even in the absence of active infection. Exclusion criteria were advanced pregnancy that could limit the exploration of the abdomen by US, and known acute (e.g. diverticulitis) or chronic inflammatory bowel diseases (e.g. Crohn disease or ulcerative rectocolitis). During the routine screening visit, all individuals underwent, among other exams, a complete blood cells count, and urine and faecal parasitology. Copromicroscopy after formol-ether concentration was performed for intestinal parasites and PCR for the detection of *Schistosoma* spp DNA in stool (modified from [14]). Urine samples were collected between 10 am and 2 pm, concentrated by filtration, and microscopically evaluated for the presence of *S*. *haematobium* eggs and for the performance of *Schistosoma* antigen CCA urinary ICT (nal von Minden GmbH, Moer, Germany). Anti-*Schistosoma* serology was performed by ELISA (Bordier Affinity Products, Crissier, Switzerland) and/or IgM-IgG immunochromatographic (ICT) test (LDBio Diagnostics, Lyon, France), according to the manufacturer’s instructions. On the same day of the visit, i.e. before laboratory results were known, individuals confirming their participation to the study by signing the informed consent form were investigated by abdominal US (all performed by FT) using a SonoSite M-Turbo (Fijifilm, Washinghton, USA) equipped with convex and linear probes. For the intestinal US investigation, the sigma, proximal part of ascending colon next to the ileocecal valve, and terminal ileum were visualized in the area between the iliac vessels and psoas muscle. The area of maximum gut wall thickness was visually identified in each of these segments and measured in each segment (in millimetres; primary outcome). Care was taken to avoid measurement in correspondence to semilunar folds in the colon and during peristaltic contractions in the ileum. Other abdominal features (presence of abdominal effusion, abnormally dilated bowel loops, enlarged mesenteric lymph nodes, mesenteric hypertrophy) recorded in the case report form, images and videos. Liver characteristics, including image pattern according to WHO [12], and spleen characteristics on US were also evaluated by US and recorded. The spleen size was evaluated using both bipolar length in relation to person’s height [15] and cross-section area [16], measured on a longitudinal scan passing through the splenic hilum.

Patients with a diagnosis of *S*. *mansoni* infection confirmed by microscopy and/or PCR were re-evaluated by intestinal US one month after completion of therapy with praziquantel (40 mg/kg/day in 2 divided doses for 3 days). This time point was decided based on experience with inflammatory lesions of the bladder mucosa in patients with *S*. *haematobium* infection, which either disappear or decrease in size within one month post-therapy in most cases [17–19]. Inflammatory indexes (Erythrocyte Sedimentation Rate [ESR, normal value <34 mm/H] and/or C Reactive Protein [CRP, normal value <5 mg/l]) were assessed as part of routine tests, while signs and symptoms at the time of hospitalization for treatment were obtained from clinical documentation.

### Statistical analysis

Individuals were classified as follows: 1) patients with *S*. *mansoni* infection confirmed by presence of *S*. *mansoni* eggs in faeces; 2) patients with *S*. *haematobium* infection confirmed by presence of S. *haematobium* eggs in urine; 3) serological schistosomiasis assessed by at least 1 positive serology test but no detection of eggs in faeces and/or urine; 4) schistosomiasis negative individuals, with no laboratory sign of infection. Patients with confirmed *S*. *mansoni* infection were analysed as the cases, while the other groups were considered as controls. Presence of other intestinal parasitic infections was considered a confounding factor and its distribution among groups evaluated. Unfortunately it was not possible to stratify results according to infection intensity, which can be a potential effect modifier, as stool examination by Kato-katz, a poorly sensitive technique not useful for diagnosis at individual patient’s level, is not part of the routine coproparasitological examination of our hospital. The Shapiro-Wilk test was used to assess the normal distribution of the data. Continuous variables were described by mean and standard deviation (SD) and compared by ANOVA in case of independent groups (case-control study) and by paired T-test in case of paired measures (longitudinal study) respectively. Categorical variables were described by count and percentage and compared by Chi-square test. Analyses were performed by MedCalc software (Ostend, Belgium). A p-value ≤ 0.05 was considered statistically significant.

## Results

### Participants characteristics

A total of 107 subjects were eligible and they all accepted to take part to the study. The details of included patients are summarized in Fig 1. No potentially eligible subject by epidemiological risk had to be excluded due to advanced pregnancy or inflammatory bowel diseases. The countries of origin of the subjects were: Nigeria (n = 24), Mali (n = 11), Côte d’Ivoire (n = 10), Senegal (n = 10), Angola (n = 8), Republic of Guinea (Guinea Conakry n = 7), the Gambia (n = 7), Ghana (n = 6), Eritrea (n = 5), Guinea Bissau (n = 3), Burkina Faso (n = 3), Democratic Republic of the Congo (n = 2), Sierra Leone (n = 2), and Brazil, Cameroon, Italy, Madagascar, Niger, Somalia, Togo, Uganda (n = 1 each). The Italian patient had probably acquired *S*. *mansoni* infection in Uganda.

One patient had concomitant *S*. *mansoni* and *S*. *haematobium* infection, and was classified in the *S*. *mansoni* group for the analysis. Among the 17 patients diagnosed with *S*. *mansoni* infection, 16 (94.1%) had positive faecal microscopy, while one (5.88%) was only positive at stool PCR; of patients with positive faecal microscopy, 13/16 (81.25%) had also positive PCR, while in 3/16 (18.75%) stool PCR was negative All patients with confirmed *S*. *haematobium* infection were diagnosed by positive microscopy of urine collected around midday, with the exception of one patient who had negative urine microscopy but presence of parasite eggs in a biopsy of the urinary bladder mucosa performed in another hospital, because of haematuria. Among the 31 patients classified as affected by schistosomiasis by serology only, ELISA was performed in 27 cases, of which 19 (70.37%) were positive, and IgM-IgG ICT was performed in 22 cases, of which 21 (95.45%) were positive. A discordant result of negative ELISA and positive ICT was observed in 8 (44.44%) of the 18 cases where both tests were applied, while the opposite was only observed in one (5.55%) of the 18 cases. Urine CCA antigen test was performed on a total of 66 individuals, with positive results only in 5 patients with *S*. *mansoni* infection, in the patient with both *S*. *haematobium* and *S*. *mansoni* infections, and in 2 patients with serological positivity but no parasitological confirmation.

### Cross-sectional study

**Fig 2:**
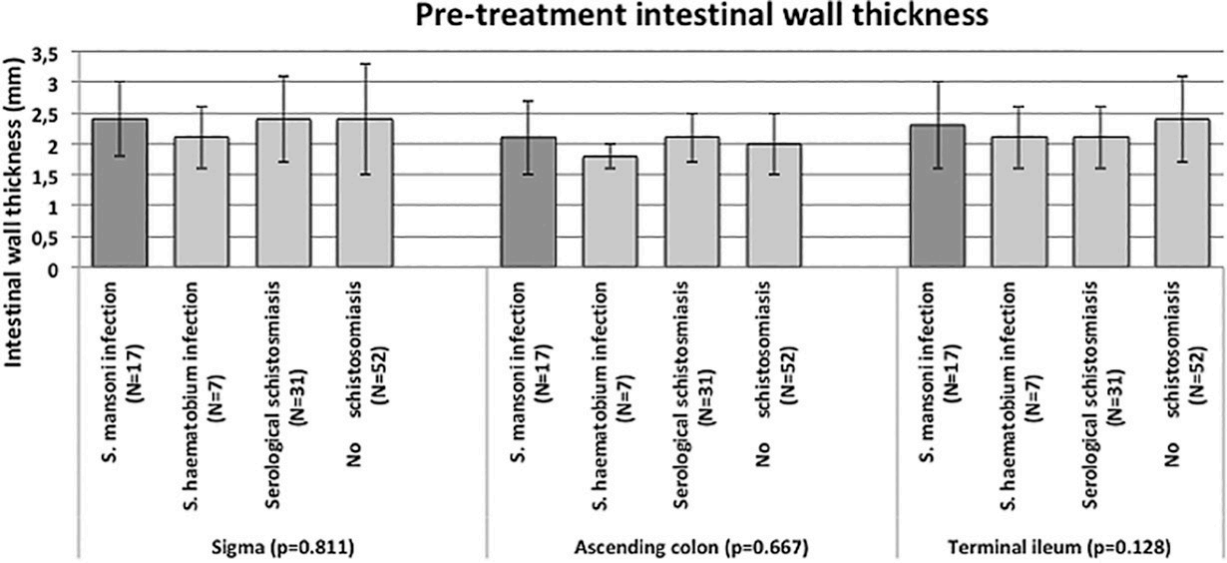
Results of the case-control study. Comparison of wall thickness of the investigated intestinal segments in *S*. *mansoni* infected cases (dark grey bars) and control groups (light grey bars). Bars indicate mean values with standard deviations. P-values refer to ANOVA statistical test.

As shown in Fig 2, there was no overtly pathological thickness (>4 mm [20]) of the intestinal wall in investigated areas in patients with *S*. *mansoni* infection. No significant differences were found with other groups for any of the investigated intestinal segments: sigma (p = 0.811); ascending colon (p = 0.667); and terminal ileum (p = 0.128). No other pathological abdominal image could be detected by US in patients with confirmed *S*. *mansoni* infection or in the other groups.

Concomitant intestinal parasitic infections, assessed by copromicroscopical exam, were present in 63 (58.88%) subjects, with no significant difference between groups (p = 0.077; Fig 1). These included various combinations of infections with *Ascaris lumbricoides*, *Blastocysts*, *Chilomastix mesnili*, *Endolimax nana*, *Entamoeba coli*, *Entamoeba hartmannii*, *Entamoeba histolytica/dispar* complex, *Entamoeba polecki*, *Giardia duodenalis*, hookworm, *Hymenolepis nana*, *Iodamoeba buetschli*, *Strongyloides stercoralis*.

### Other characteristics of *S*. *mansoni* infected patients and longitudinal study

At diagnosis, no *S*. *mansoni* infected patient had US signs of hepatic portal fibrosis, with the exception of two patients showing a pattern C (“ring echoes and pipe stem” [12]), one of whom also had a “starry sky” appearance of a normal sized spleen. Mild to moderate splenomegaly was observed in 6 patients with *S*. *mansoni* infection when the cross-sectional area was used (mean spleen area in splenomegalic patients = 67.33 cm^2^, SD 20.28), while also bipolar size in relation to height was above normal limits in 2 of these patients (15.6 cm and 13 cm in male patients 175 cm and 165 cm tall, respectively). During hospitalization, no blood nor mucus in stool was observed in patients infected with *S*. *mansoni*; 3 patients had slight pain to the palpation of the abdomen. Three patients referred “abdominal pain” either presently or in the past, and 2 reported “occasional red blood in stool in the past”. However, the cultural and linguistic barriers often present limit the reliability of these data.

ESR was within normal limits in all patients (mean 11 mm/H, SD 6); CRP was normal (1.37 mg/l, SD 0.61) in all patients with the exception of one (8.81 mg/l) who also had splenomegaly and abdominal pain.

**Fig 3:**
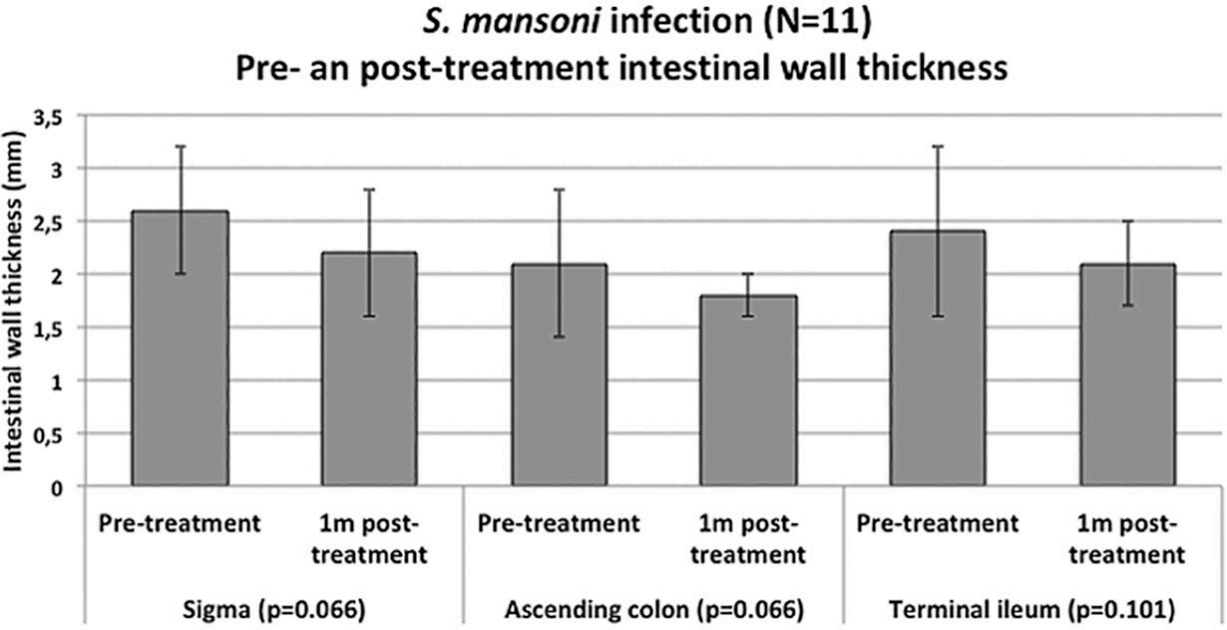
Results of the longitudinal study. Comparison of wall thickness of the investigated intestinal segments in *S*. *mansoni* infected at the time of diagnosis (pre-treatment) and 1 month post-treatment with praziquantel. Bars indicate mean values with standard deviations. P-values refer to paired T-test statistical test.

The maximum intestinal wall thickness in each investigated segment was measured one month after treatment with praziquantel in 11 out of the 17 patients with *S*. *mansoni* infection. There was no significant change in maximum wall thickness of the sigma (p = 0.066), ascending colon (p = 0.066), and terminal ileum (p = 0.101) one month after treatment (Fig 3)

## Discussion

The involvement of the intestine in pathology due to *S*. *mansoni* infection, due to the route of elimination of parasite eggs, is documented by histological and endoscopic findings [21–23]. It is also reflected in the association between positivity of faecal occult blood and calprotectin and medium to high egg intensity output in intestinal schistosomiasis [9, 24, 25], and between schistosomiasis and anaemia [26]. The prompt response to praziquantel of faecal occult blood and calprotectin most likely indicates rapid mucosal integrity restoration after treatment [9]. Although these laboratory tests could be used as markers of *S*. *mansoni* intestinal morbidity, non-invasive assessment of intestinal pathology, as it could be provided by US, would allow a more comprehensive assessment of morbidity before and after treatment at both individual patient level in the clinical setting and in community studies. US reports on intestinal schistosomiasis, however, are extremely scant. Dittrich and colleagues [13] performed intestinal US on 173 individuals in an endemic area of Senegal, reporting intestinal abnormalities in 82% of patients with documented (Kato-Katz positive) *S*. *mansoni* infection and 60% of uninfected patients, with an apparent positive correlation between infection intensity (eggs per gram of faeces) and prevalence of abnormalities on US. These included intestinal wall thickening, pseudo-kidney or cockade-like images, and increased echogenicity of the mucosa and of the mesenteric structures. None of these abnormalities were detected in our cohort. Many factors (endemic vs non-endemic area, inclusion vs exclusion of children, different intensity of infections in the two cohorts, different coproparasitological diagnostic method used, prevalence and type of concomitant intestinal infections, image resolution of the US machine and of the probe used–convex low-frequency probe by Dittrich and colleagues vs linear high-frequency highly accurate probe in our study) could have contributed to the very different results of the two studies. Also, the reported very high prevalence of abnormal intestinal findings in individuals apparently not infected with *S*. *mansoni* supports the hypothesis that the two study populations were markedly different, and therefore difficult to compare. However, a common finding was the lack of visualization of polypoid formations, which conversely are detected in infected patients by colonoscopy [22]. This is consistent with the very low sensitivity of unenhanced US for this type of lesions. Sensitivity could be increased by the use of hydrocolonic sonography [27], however, this technique is time consuming, generally poorly accepted, and not suitable for large-scale studies.

The absence of visualization of intestinal wall thickening in our cohort of patients eliminating *S*. *mansoni* eggs in faeces was surprising, although this result should be considered preliminary due to the small sample size. The absence of difference between cases and controls in intestinal wall thickness, with this being overall within normal limits in all groups, counters the possible bias deriving from including *S*. *mansoni* infected individuals in other control groups due to low sensitivity of diagnostic methods. It is possible to speculate that only minimal inflammation was expressed in the examined patients. This is supported by the normal systemic inflammation markers in almost all patients, as well as by the absence or low prevalence of clear signs of infection at diagnosis and reported current or past signs and symptoms. These, however, are known to have low sensitivity in infected individuals [28] and collection of an accurate clinical history was often hampered by cultural and linguistic barriers. Unfortunately local markers such as faecal occult blood and calprotectin are not routinely tested in patients diagnosed with schistosomiasis in our centre, and were therefore not available. Another limitation of our study is the unknown intensity of infection in terms of eggs per gram of faeces, as routine copromicroscopical examination in our centre is carried out using formol-ether concentration and not Kato-Katz. Several epidemiological and intervention studies found a correlation between *S*. *mansoni* infection intensity and morbidity, including portal fibrosis [29–34]. Considering the overall clinical picture of the patients involved in our cohort, it is therefore plausible that our cohort of *S*. *mansoni* infected patients may have included only subjects with low intensity infections.

In conclusion, we carried out for the first time a pilot, prospective case-control study of intestinal unenhanced US for *S*. *mansoni* morbidity assessment outside endemic areas, using a standard protocol and high-resolution US equipment. Our preliminary results suggest that, although appealing, intestinal US is not likely to be a useful tool in all settings for a more comprehensive assessment of morbidity before and after treatment for *S*. *mansoni* infection. Further studies are needed to describe and assess the usefulness of intestinal US on different populations, such as in endemic areas and in patients stratified by infection intensity. Ultrasound findings may be compared with the results of fecal calprotectin and fecal occult blood tests [9]. In the hospital setting, studies comparing colonoscopy and ultrasonography could be envisaged.

## Supporting information

S1 Table Individual subjects data. (XLSX)

S2 Table STROBE checklist for case-control studies. (DOC)

## References

[1] Schistosomiasis and soil-transmitted helminthiases: number of people treated in 2016. Wkly Epidemiol Rec. 2017;92(49):749–60. 29218962

[2] Steinmann P, Keiser J, Bos R, Tanner M, Utzinger J. Schistosomiasis and water resources development: systematic review, meta-analysis, and estimates of people at risk. Lancet Infect Dis. 2006;6(7):411–25. 10.1016/S1473-3099(06)70521-7 16790382

[3] Beltrame A, Buonfrate D, Gobbi F, Angheben A, Marchese V, Monteiro GB, et al The hidden epidemic of schistosomiasis in recent African immigrants and asylum seekers to Italy. Eur J Epidemiol. 2017. 10.1007/s10654-017-0259-6 28560535

[4] van der Werf MJ, de Vlas SJ, Brooker S, Looman CW, Nagelkerke NJ, Habbema JD, et al Quantification of clinical morbidity associated with schistosome infection in sub-Saharan Africa. Acta Trop. 2003;86(2–3):125–39. 12745133 10.1016/s0001-706x(03)00029-9

[5] Colley DG, Bustinduy AL, Secor WE, King CH. Human schistosomiasis. Lancet. 2014;383(9936):2253–64. 10.1016/S0140-6736(13)61949-2 24698483 PMC4672382

[6] el Scheich T, Holtfreter MC, Ekamp H, Singh DD, Mota R, Hatz C, et al The WHO ultrasonography protocol for assessing hepatic morbidity due to Schistosoma mansoni. Acceptance and evolution over 12 years. Parasitol Res. 2014;113(11):3915–25. 10.1007/s00436-014-4117-0 25260691

[7] French MD, Evans D, Fleming FM, Secor WE, Biritwum NK, Brooker SJ, et al Schistosomiasis in Africa: Improving strategies for long-term and sustainable morbidity control. PLoS Negl Trop Dis. 2018;12(6):e0006484 10.1371/journal.pntd.0006484 29953454 PMC6023105

[8] Richter J, Correia Dacal AR, Vergetti Siqueira JG, Poggensee G, Mannsmann U, Deelder A, et al Sonographic prediction of variceal bleeding in patients with liver fibrosis due to Schistosoma mansoni. Trop Med Int Health. 1998;3(9):728–35. 9754668 10.1046/j.1365-3156.1998.00285.x

[9] Bustinduy AL, Sousa-Figueiredo JC, Adriko M, Betson M, Fenwick A, Kabatereine N, et al Fecal occult blood and fecal calprotectin as point-of-care markers of intestinal morbidity in Ugandan children with Schistosoma mansoni infection. PLoS Negl Trop Dis. 2013;7(11):e2542 10.1371/journal.pntd.0002542 24244777 PMC3828154

[10] Belard S, Tamarozzi F, Bustinduy AL, Wallrauch C, Grobusch MP, Kuhn W, et al Point-of-Care Ultrasound Assessment of Tropical Infectious Diseases—A Review of Applications and Perspectives. Am J Trop Med Hyg. 2016;94(1):8–21. 10.4269/ajtmh.15-0421 26416111 PMC4710450

[11] Richter J, Holtfreter M, Mouahid G, Mone H. Acute anuria after a family vacation to Corsica/France. Parasitol Res. 2016;115(4):1733–5. 10.1007/s00436-016-4944-2 26852123

[12] W.H.O. Ultrasound in schistosomiasis: a practical guide to the standard use of ultrasonography for assessment of schistosomiasis-related morbidity. 2000 http://wwwwhoint/schistosomiasis/resources/tdr_str_sch_001/en/ Accessed on October 2017.

[13] Dittrich M, Thomas AK, Stelma FF, Talla I, Niang M, Decam C, et al Preliminary ultrasonographical observations of intestinal lesions in a community with heavy Schistosoma mansoni infection in Richard Toll, Senegal. Acta Trop. 1994;58(3–4):331–6. 7709871 10.1016/0001-706x(94)90026-4

[14] Obeng BB, Aryeetey YA, de Dood CJ, Amoah AS, Larbi IA, Deelder AM, et al Application of a circulating-cathodic-antigen (CCA) strip test and real-time PCR, in comparison with microscopy, for the detection of Schistosoma haematobium in urine samples from Ghana. Ann Trop Med Parasitol. 2008;102(7):625–33. 10.1179/136485908X337490 18817603

[15] Chow KU, Luxembourg B, Seifried E, Bonig H. Spleen Size Is Significantly Influenced by Body Height and Sex: Establishment of Normal Values for Spleen Size at US with a Cohort of 1200 Healthy Individuals. Radiology. 2016;279(1):306–13. 10.1148/radiol.2015150887 26509293

[16] Ishibashi H, Higuchi N, Shimamura R, Hirata Y, Kudo J, Niho Y. Sonographic assessment and grading of spleen size. J Clin Ultrasound. 1991;19(1):21–5. 1846375 10.1002/jcu.1870190106

[17] Hatz C, Mayombana C, de Savigny D, MacPherson CN, Koella JC, Degremont A, et al Ultrasound scanning for detecting morbidity due to Schistosoma haematobium and its resolution following treatment with different doses of praziquantel. Trans R Soc Trop Med Hyg. 1990;84(1):84–8. 2111949 10.1016/0035-9203(90)90392-r

[18] Hatz CF, Vennervald BJ, Nkulila T, Vounatsou P, Kombe Y, Mayombana C, et al Evolution of Schistosoma haematobium-related pathology over 24 months after treatment with praziquantel among school children in southeastern Tanzania. Am J Trop Med Hyg. 1998;59(5):775–81. 9840596 10.4269/ajtmh.1998.59.775

[19] Wagatsuma Y, Aryeetey ME, Sack DA, Morrow RH, Hatz C, Kojima S. Resolution and resurgence of schistosoma haematobium-induced pathology after community-based chemotherapy in ghana, as detected by ultrasound. J Infect Dis. 1999;179(6):1515–22. 10.1086/314786 10228074

[20] Fleischer AC, Muhletaler CA, James AE Jr. Sonographic assessment of the bowel wall. AJR Am J Roentgenol. 1981;136(5):887–91. 10.2214/ajr.136.5.887 6784522

[21] Cao J, Liu WJ, Xu XY, Zou XP. Endoscopic findings and clinicopathologic characteristics of colonic schistosomiasis: a report of 46 cases. World J Gastroenterol. 2010;16(6):723–7. 10.3748/wjg.v16.i6.723 20135720 PMC2817060

[22] El-Shabrawi MH, El Din ZE, Isa M, Kamal N, Hassanin F, El-Koofy N, et al Colorectal polyps: a frequently-missed cause of rectal bleeding in Egyptian children. Ann Trop Paediatr. 2011;31(3):213–8. 10.1179/1465328111Y.0000000014 21781415

[23] Saad AM, el Masri SH, Omer AH, el Hassan AM. A clinico-pathological study on intestinal bilharziasis in the Sudan. East Afr Med J. 1985;62(6):397–402. 3930203

[24] Kanzaria HK, Acosta LP, Langdon GC, Manalo DL, Olveda RM, McGarvey ST, et al Schistosoma japonicum and occult blood loss in endemic villages in Leyte, the Philippines. Am J Trop Med Hyg. 2005;72(2):115–8. 15741543

[25] Ndamba J, Makaza N, Kaondera KC, Munjoma M. Morbidity due to Schistosoma mansoni among sugar-cane cutters in Zimbabwe. Int J Epidemiol. 1991;20(3):787–95. 1955265 10.1093/ije/20.3.787

[26] Friedman JF, Kanzaria HK, McGarvey ST. Human schistosomiasis and anemia: the relationship and potential mechanisms. Trends Parasitol. 2005;21(8):386–92. 10.1016/j.pt.2005.06.006 15967725

[27] Kuzmich S, Harvey CJ, Kuzmich T, Tan KL. Ultrasound detection of colonic polyps: perspective. Br J Radiol. 2012;85(1019):e1155–64. 10.1259/bjr/60593124 22806624 PMC3500816

[28] Lengeler C, Utzinger J, Tanner M. Screening for schistosomiasis with questionnaires. Trends Parasitol. 2002;18(9):375–7. 12377245 10.1016/s1471-4922(02)02318-8

[29] Andrade G, Bertsch DJ, Gazzinelli A, King CH. Decline in infection-related morbidities following drug-mediated reductions in the intensity of Schistosoma infection: A systematic review and meta-analysis. PLoS Negl Trop Dis. 2017;11(2):e0005372 10.1371/journal.pntd.0005372 28212414 PMC5333910

[30] Berhe N, Myrvang B, Gundersen SG. Intensity of Schistosoma mansoni, hepatitis B, age, and sex predict levels of hepatic periportal thickening/fibrosis (PPT/F): a large-scale community-based study in Ethiopia. Am J Trop Med Hyg. 2007;77(6):1079–86. 18165526

[31] King CH, Magak P, Salam EA, Ouma JH, Kariuki HC, Blanton RE, et al Measuring morbidity in schistosomiasis mansoni: relationship between image pattern, portal vein diameter and portal branch thickness in large-scale surveys using new WHO coding guidelines for ultrasound in schistosomiasis. Trop Med Int Health. 2003;8(2):109–17. 12581434 10.1046/j.1365-3156.2003.00994.x

[32] Malenganisho WL, Magnussen P, Friis H, Siza J, Kaatano G, Temu M, et al Schistosoma mansoni morbidity among adults in two villages along Lake Victoria shores in Mwanza District, Tanzania. Trans R Soc Trop Med Hyg. 2008;102(6):532–41. 10.1016/j.trstmh.2008.03.006 18440577

[33] Mohamed-Ali Q, Elwali NE, Abdelhameed AA, Mergani A, Rahoud S, Elagib KE, et al Susceptibility to periportal (Symmers) fibrosis in human schistosoma mansoni infections: evidence that intensity and duration of infection, gender, and inherited factors are critical in disease progression. J Infect Dis. 1999;180(4):1298–306. 10.1086/314999 10479161

[34] Tukahebwa EM, Magnussen P, Madsen H, Kabatereine NB, Nuwaha F, Wilson S, et al A very high infection intensity of Schistosoma mansoni in a Ugandan Lake Victoria Fishing Community is required for association with highly prevalent organ related morbidity. PLoS Negl Trop Dis. 2013;7(7):e2268 10.1371/journal.pntd.0002268 23936559 PMC3723538

