# Supplementary material for: Ultrasound and intestinal lesions in Schistosoma mansoni infection: A case-control pilot study outside endemic areas
Source: PLoS One. 2018 Dec 18;13(12):e0209333. doi: 10.1371/journal.pone.0209333 (PMC6298661; doi:10.1371/journal.pone.0209333)
Supplement: S2 Table — (DOC) [file pone.0209333.s002.doc]

STROBE Statement—Checklist of items that should be included in reports of ***case-control studies***

|  | Item No | Recommendation |
| --- | --- | --- |
| **Title and abstract** | 1 | (*a*) Indicate the study’s design with a commonly used term in the title or the abstract  “Case-control pilot study” is indicated in the title |
|  |  | (*b*) Provide in the abstract an informative and balanced summary of what was done and what was found  “Methodology/Principal findings” in the Abstract |
| Introduction | | |
| Background/rationale | 2 | Explain the scientific background and rationale for the investigation being reported  “Introduction” in particular second and third paragraph |
| Objectives | 3 | State specific objectives, including any pre-specified hypotheses  “Introduction” last paragraph |
| Methods | | |
| Study design | 4 | Present key elements of study design early in the paper  “Methods”, “Study design and participants” section, first sentence |
| Setting | 5 | Describe the setting, locations, and relevant dates, including periods of recruitment, exposure, follow-up, and data collection  “Methods”, “Study design and participants” section, first paragraph |
| Participants | 6 | (*a*) Give the eligibility criteria, and the sources and methods of case ascertainment and control selection. Give the rationale for the choice of cases and controls  “Methods”, “Study design and participants” section, second paragraph, and “Methods”, Statistical analysis” section, first paragraph. |
|  |  | (*b*) For matched studies, give matching criteria and the number of controls per case  N/A |
| Variables | 7 | Clearly define all outcomes, exposures, predictors, potential confounders, and effect modifiers. Give diagnostic criteria, if applicable  “Methods”, “Study design and participants” section, second paragraph, and “Methods”, Statistical analysis” section, first paragraph. |
| Data sources/ measurement | 8* | For each variable of interest, give sources of data and details of methods of assessment (measurement). Describe comparability of assessment methods if there is more than one group  “Methods”, “Study design and participants” section, second paragraph |
| Bias | 9 | Describe any efforts to address potential sources of bias  N/A in the “Methods”, but addressed in the “Discussion”, beginning of second paragraph |
| Study size | 10 | Explain how the study size was arrived at  “Methods”, first sentence |
| Quantitative variables | 11 | Explain how quantitative variables were handled in the analyses. If applicable, describe which groupings were chosen and why  Methods”, Statistical analysis” section, first and second paragraph |
| Statistical methods | 12 | (*a*) Describe all statistical methods, including those used to control for confounding  Methods”, Statistical analysis” section, second paragraph |
|  |  | (*b*) Describe any methods used to examine subgroups and interactions  N/A |
|  |  | (*c*) Explain how missing data were addressed  N/A |
|  |  | (*d*) If applicable, explain how matching of cases and controls was addressed  N/A |
|  |  | (*e*) Describe any sensitivity analyses  N/A |
| Results | | |
| Participants | 13* | (a) Report numbers of individuals at each stage of study—eg numbers potentially eligible, examined for eligibility, confirmed eligible, included in the study, completing follow-up, and analysed  “Results”, “Participants characteristics”, and “Other *S. mansoni* infected patients characteristics and longitudinal study”; AND Figure 1 |
|  |  | (b) Give reasons for non-participation at each stage  N/A |
|  |  | (c) Consider use of a flow diagram  Figure 1 |
| Descriptive data | 14* | (a) Give characteristics of study participants (eg demographic, clinical, social) and information on exposures and potential confounders  “Results”, “Participants characteristics”, and “Cross-sectional study”; AND Figure 1 |
|  |  | (b) Indicate number of participants with missing data for each variable of interest  N/A |
| Outcome data | 15* | Report numbers in each exposure category, or summary measures of exposure  “Results”, “Cross-sectional study” and “Other *S. mansoni* infected patients characteristics and longitudinal study”; AND Figures 2 and 3 |
| Main results | 16 | (*a*) Give unadjusted estimates and, if applicable, confounder-adjusted estimates and their precision (eg, 95% confidence interval). Make clear which confounders were adjusted for and why they were included  Figures 2 and 3 |
|  |  | (*b*) Report category boundaries when continuous variables were categorized  N/A |
|  |  | (*c*) If relevant, consider translating estimates of relative risk into absolute risk for a meaningful time period  N/A |

| Other analyses | 17 | Report other analyses done—eg analyses of subgroups and interactions, and sensitivity analyses  N/A |
| --- | --- | --- |
| Discussion | | |
| Key results | 18 | Summarise key results with reference to study objectives  “Discussion”, second and third paragraphs |
| Limitations | 19 | Discuss limitations of the study, taking into account sources of potential bias or imprecision. Discuss both direction and magnitude of any potential bias  “Discussion”, second and third paragraphs |
| Interpretation | 20 | Give a cautious overall interpretation of results considering objectives, limitations, multiplicity of analyses, results from similar studies, and other relevant evidence  “Discussion”, first, second, paragraphs but in particular third paragraphs |
| Generalisability | 21 | Discuss the generalisability (external validity) of the study results  “Discussion”, third paragraph |
| Other information | | |
| Funding | 22 | Give the source of funding and the role of the funders for the present study and, if applicable, for the original study on which the present article is based  Provided through online submission system of the journal |

*Give information separately for cases and controls.

**Note:** An Explanation and Elaboration article discusses each checklist item and gives methodological background and published examples of transparent reporting. The STROBE checklist is best used in conjunction with this article (freely available on the Web sites of PLoS Medicine at http://www.plosmedicine.org/, Annals of Internal Medicine at http://www.annals.org/, and Epidemiology at http://www.epidem.com/). Information on the STROBE Initiative is available at http://www.strobe-statement.org.
